# Biglycan fragment modulates TGF-β activity in intervertebral disc via an eIF6-coupled intracellular path

**DOI:** 10.1126/sciadv.adq8545

**Published:** 2025-02-14

**Authors:** Manyu Zhu, Stanley Chun Ming Wu, Wai-Kit Tam, Chun Kit Wong, Peng Liao, Kathryn S. Cheah, Danny Chan, Aaron W. James, Victor Y. Leung

**Affiliations:** ^1^ Department of Orthopaedics and Traumatology, School of Clinical Medicine, The University of Hong Kong, Hong Kong SAR, China.; ^2^ School of Biomedical Sciences, The University of Hong Kong, Hong Kong SAR, China.; ^3^ Department of Pathology, Johns Hopkins University, Baltimore, MD 21205, USA.; ^4^ Department of Biomedical Engineering, Johns Hopkins University, Baltimore, MD 21205, USA.

## Abstract

Biglycan, a pericellular small leucine-rich proteoglycan, is crucial in skeletal development and regeneration. Intervertebral disc degeneration (IDD) contributes to back pain and disability. Previous studies have shown that biglycan promotes hypoxic survival of disc progenitor cells, while its depletion accelerates IDD. An association of pathological tissue remodeling with a biglycan fragment ^344^YWEVQPATFR, termed Bgm1, has been reported, however its role is yet to be defined. Using a custom antibody, we detected Bgm1 in human and mouse nucleus pulposus, with prominent intracellular expression in notochordal cells. Proteomic analysis revealed that Bgm1 interacts with eukaryotic translation initiation factor 6 (eIF6), a key player in ribosome biogenesis. Bgm1 dysregulates eIF6 localization in notochordal cells, affecting nucleocytoplasmic transport. Induced IDD in mice showed elevated nuclear eIF6 expression and reduced Bgm1 in degenerating nucleus pulposus. Transcriptome analysis suggests that Bgm1 regulates fatty acid metabolism and glycolysis in a transforming growth factor–β–dependent manner, highlighting its potential role in metabolic control in spinal joint homeostasis.

## Teaser

An intracellular biglycan fragment partners with eIF6, a ribosome biogenesis and metabolic regulator to preserve disc homeostasis.

## INTRODUCTION

Intervertebral disc (IVD) degeneration (IDD) is one of the major age-related spine disorders, with 31% of young adults being diagnosed in their 20s (*1*). IDD causes neck and back pain, which severely affects the quality of life and has become a top global disease burden in the aging population (*2*, *3*). Understanding the mechanism of IDD is therefore important to future management of the disease burden.

As a proteoglycan-rich gelatinous core, the nucleus pulposus (NP) confers mechanical strength and shock absorption to the disc under axial loads (*4*). IDD is linked to chronic inflammation, which leads to excessive degradation and fibrous remodeling of the extracellular matrix (ECM) in the NP (*4*, *5*). Biologics that enhance ECM synthesis or reduce inflammation have been shown to alleviate IDD (*6*). NP cells (NPCs) are derived from the notochord, and because of the avascular nature of the disc, they rely on anaerobic glycolysis for energy and unique metabolic control to survive and function (*7*, *8*). NPCs produce proteoglycans and collagens, in particular aggrecan and collagen II (*9*), as well as cytokines such as interleukin-1 (IL-1), IL-6, and IL-10 (*10*) and matrix metalloproteinases (MMPs) (*11*), to modulate the IVD function. IDD is associated with the loss, senescence, and altered function of the NPCs (*12*). Because the impaired disc cannot withstand normal loads, nearby supportive structures, especially the ligaments and muscles, can also be affected. These structures compensate for the weakened disc mechanics and experience increased stress because of altered biomechanics and reduced shock absorption (*13*, *14*).

Biglycan is a class I small leucine-rich proteoglycan (SLRP) and comprises a core leucine-rich repeats region flanked by cysteine residues and one or two chondroitin sulfate/dermatan sulfate glycosaminoglycan side chains bound covalently through a tetrasaccharide bridge to a serine residue (*15*). It has been considered as a matricellular protein because of its extracellular expression and ability to regulate cell-matrix cross-talk both directly (through receptors) and indirectly (by interactions with cytokines, growth hormones, and other ECM components) (*16*). Sequestered biglycan can deposit in the ECM to promote fibrosis or scar formation (*17*), whereas nonsequestered, soluble biglycan can be released from the ECM as a signaling factor in the inflammatory response (*18*). Deglycosylated biglycan may promote bone formation and regeneration by activating bone morphogenetic protein signaling (*19*, *20*). Degradation of biglycan may contribute to ECM remodeling–related diseases such as osteoarthritis, rheumatoid arthritis, and IDD (*15*, *21*).

Fragmented species of biglycan in disc specimens from both scoliotic and IDD subjects and their changes with age have been reported (*22*, *23*). The knockout of biglycan in mice can accelerate IDD with abnormal disc tissue remodeling (*15*, *24*). Biglycan can promote disc progenitor survival under hypoxic stress (*25*). These indicate that biglycan is essential to IVD homeostasis. A previous study reported a highly conserved C-terminal neoepitope ^344^YWEVQPATFR, termed Bgm1, which could be cleaved by MMP12 and released from an ex vivo bovine cartilage explant culture as a potential marker of matrix turnover (*26*). Whether Bgm1 has a biological function and has a role in health and diseases remains elusive. Our previous study identified an up-regulation of MMP12 and myofibroblast markers in human and rodent IDD (*27*). Given the expression of biglycan in the IVD and that its loss cause IDD, we hypothesized that biglycan fragmentation may have a role in regulating IVD homeostasis. In this study, we report dynamic changes of Bgm1 expression in normal IVD and in IDD. Results from transcriptomic and proteomic studies suggest that Bgm1 can have an intracellular role in regulating NPC metabolism via an interaction with eukaryotic translation initiation factor 6 (eIF6).

## RESULTS

### Bgm1 production marks healthy NP and reduces in IDD

**Fig. 1.**
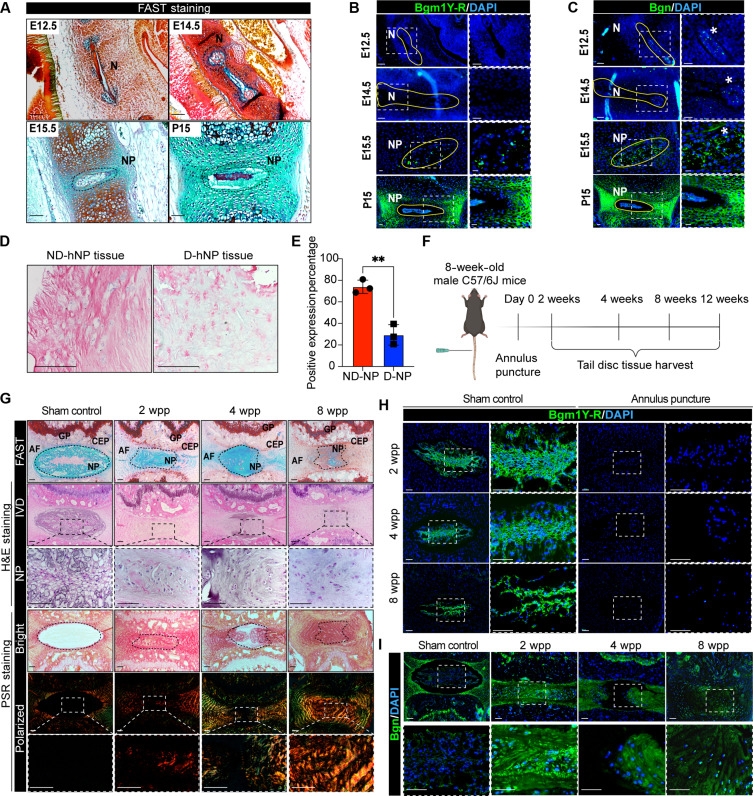
Bgm1 expression pattern in IVD development and degeneration. (**A**) FAST staining of embryonic and early postnatal mouse IVDs. (**B** and **C**) Bgn and Bgm1Y-R immunofluorescence staining of embryonic and early postnatal mouse IVDs. (**D**) Representative images showing immunohistochemistry staining for Bgm1 in human NP tissues. The Bgm1-positive signal was revealed by alkaline phosphatase (pink). (**E**) Quantification of Bgm1 staining shown as the percentage of positive expression region in human NP tissues (*n* = 3). (**F**) Schematic diagram of the puncture-induced IDD mouse models. (**G**) Representative image of FAST staining, H&E staining, and PSR staining of annulus puncture–induced degenerative mouse discs. (**H** and **I**) Bgm1Y-R (H) and Bgn (I) immunofluorescence in the puncture-induced degenerative mouse discs. The dashed box shows the high magnification of the NP region. ND-hNP, nondegenerative human NP; D-hNP, degenerative human NP at grade IV or above; N, notochord; NP, nucleus pulposus; AF, annulus fibrosus; CEP, cartilaginous endplate; GP, growth plate. Scale bars, 50 μm in (D), 100 μm in (A) to (C) and (G) to (I). Data presented as means ± SD. The unpaired two-tailed Student *t* test was used for a two-group comparison. **P < 0.01.

To examine the presence and changes of Bgm1 neoepitope production in the NP development and degeneration, we performed immunostaining using a custom polyclonal antibody Bgm1Y-R targeted against the peptide. The specificity of the Bgm1Y-R antibody was validated by a detection of endogenous Bgm1 production in 293 cells but not in biglycan (BGN) knockout cells (fig. S1). The notochord develops as a rod-like structure at the midline of the fetus at embryonic day 12.5 (E12.5) and E14.5 and differentiates into the NP by E15.5 (Fig. 1A). We detected Bgm1 expression in the primitive NPCs, which is predominated by vacuolated notochordal cells, starting from E15.5 (Fig. 1B). In contrast, biglycan expression could be found in the notochord by E12.5 and later in the developed IVDs at E15.5 and neonatal stage P15 (postnatal day 15) (Fig. 1C).

We examined whether there is a difference in Bgm1 production between healthy and degenerative human discs. Immunostaining revealed a markedly reduced Bgm1 protein expression in degenerative NP samples compared to nondegenerative control (Fig. 1, D and E). We adopted a previously established mouse annulus puncture model (*10*) to further examine the Bgm1 production in the course of injury-mediated IDD and the associated remodeling events (*7*) (Fig. 1F). The induction of degeneration was confirmed by reduced Alcian blue intensity (which detects sulfated GAGs) in the NP in fast green, Alcian blue, Safranin-O, and tartrazine (FAST) staining (*28*), disappearance of the NP/annulus fibrosus (AF) border, and replacement of large vacuolated cells with chondrocyte-like cells, consistent with the characteristics of IDD (*7*) (Fig. 1G). Picrosirius red (PSR) staining indicated an increased birefringence of thick wavy collagen fibers in the NP of the punctured discs (Fig. 1G), suggesting fibrotic tissue remodeling. Bgm1 production was found to be restricted to the NP in healthy adult mice, while punctured discs exhibited a loss of expression (Fig. 1H). In contrast, biglycan production was detected throughout the entire discs under both conditions (Fig. 1I). A partial recovery from the injury was observed at 12 weeks postpuncture (wpp), as indicated by enhanced Alcian blue staining, the reappearance of vacuolated cell clusters, and a diminished collagen deposition within the NP (fig. S2A). This recovery was accompanied by Bgm1 reproduction, whereas biglycan production was reduced (fig. S2B). Together, these findings demonstrated a loss of Bgm1 production during IDD and matrix remodeling in the NP.

### Exogenous Bgm1 regulates collagen- and immunomodulation-related pathways in NPCs

**Fig. 2.**
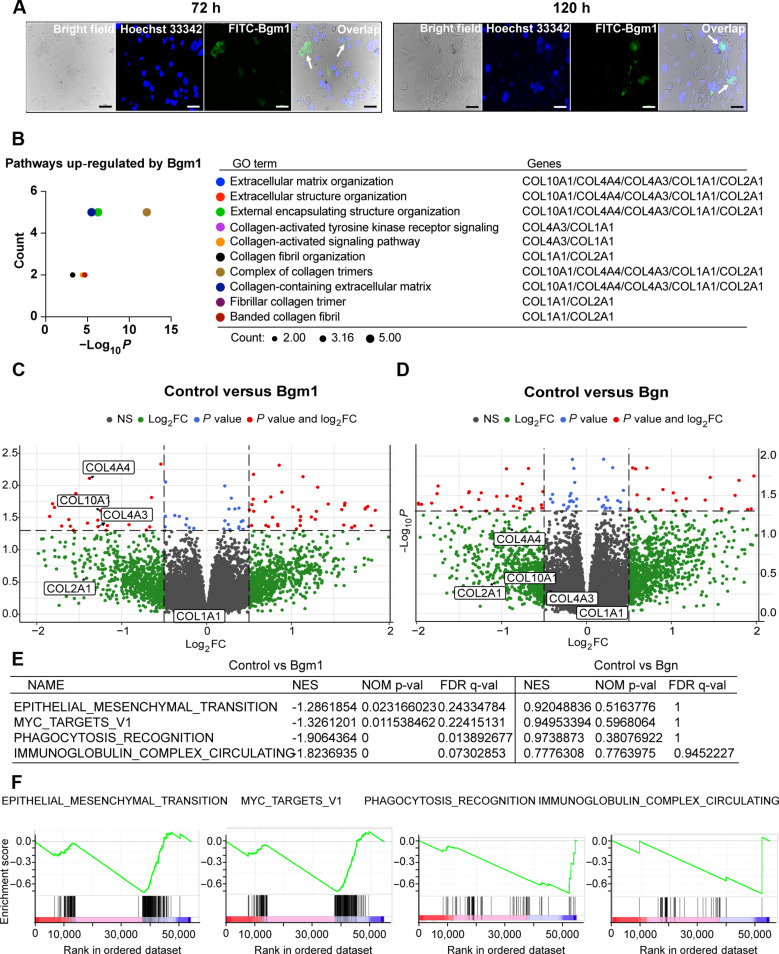
Exogenous Bgm1 stimulates matrix remodeling and immunomodulation in NPCs. (**A**) Live fluorescence images of JHC7 cells at 72 and 120 hours (h) after FITC-Bgm1 peptide treatment. Nuclei were stained with Hoechst 33342 (blue). Arrows indicate FITC-Bgm1 signal in the nucleus. (**B**) GO term analysis of DEGs significantly up-regulated by Bgm1 in the nontreatment control versus Bgm1-treatment group. The significantly enriched GO term was adjusted through *P* < 0.05. (**C** and **D**) Volcano plot of DEGs in the nontreatment control versus Bgm1 treatment group (C) and nontreatment control versus Bgn treatment group (D). The *x* axis represented log_2_ fold change (FC), and the *y* axis represented significance. Log_2_ FC cutoff 0.5 and *P* value cutoff 0.05 were used. Bgm1 and Bgn induce a negative log_2_ FC for up-regulated genes. The red color on the upper left represents genes significantly up-regulated by Bgm1 having a 0.5-fold increase, and the green area represents genes with nonsignificant expression. (**E**) GSEA of Bgm1-induced transcriptional profiles. Gene sets significantly enriched among class A (nontreated control) and class B (Bgm1-treated group) (left panel). Gene sets not significantly enriched among class A (nontreated group) and class B (Bgn-treated group) (right panel). (**F**) GSEA distribution graph of Bgm1 under normal condition. Class A: nontreated control; class B: Bgm1-treated group. The green curve represented the enrichment score of each gene corresponding to the gene set, the barcode-like black lines showed the locations of the genes in the gene set, and each vertical line represented a gene in that pathway. The enriched gene sets showed an increasing trend. Significant enrichment criterion: |NES| > 1; p-val < 0.05; FDR q-val < 0.25. NES, normalized enrichment score; NOM p-val, nominal *P* value; FDR q-val, false discovery rate. Scale bars, 100 μm.

Given that biglycan is an ECM protein, we predicted that the cellular Bgm1 signal in the NPCs is due to an uptake of the neoepitope from processed biglycan. Treatment of Johns Hopkins Chordoma line 7 (JHC7) cells with fluorescein isothiocyanate (FITC)–conjugated Bgm1 indicated Bgm1 uptake with perinuclear and nuclear localization (Fig. 2A). To gain insights into the potential physiological role of Bgm1, we incubated nondegenerative human NPCs (hND-NPCs), which show a low level of intracellular Bgm1 protein expression, with an exogenous Bgm1 peptide followed by transcriptome analysis. Gene Oncology (GO) term analyses indicated that exogenous Bgm1 notably up-regulated pathways associated with ECM organization and structural assembly (Fig. 2B). In contrast, expression of these differential genes was not changed under the treatment with equimolar full-length biglycan (Fig. 2, C and D). Moreover, Bgm1 but not full-length biglycan notably enriched genes related to the epithelial-mesenchymal transition (EMT) and Myc targets (Fig. 2E), which are known to contribute to immunosuppression and prevent cell apoptosis and senescence (Fig. 2F) (*29*, *30*). Pathways related to phagocytosis recognition and immunoglobulin complex circulation were also notably enriched (Fig. 2, E and F). Direct comparison between both peptide treatments showed that full-length biglycan significantly increased the expression of metalloproteinase *ADAM1B* and fibroblast growth factor *FGF17* (fig. S3A), whereas Bgm1 markedly down-regulated lipid-related metabolism as well as pathways related to nociception (*31*) and inflammation (*32*) (fig. S3B). These findings indicate that Bgm1 exerts distinct regulatory effects, particularly in modulating metabolic, nociceptive, and inflammatory processes, compared to full-length biglycan.

### Bgm1 attenuates ribosome and metabolic pathways induced by TGF-β1 signaling

**Fig. 3.**
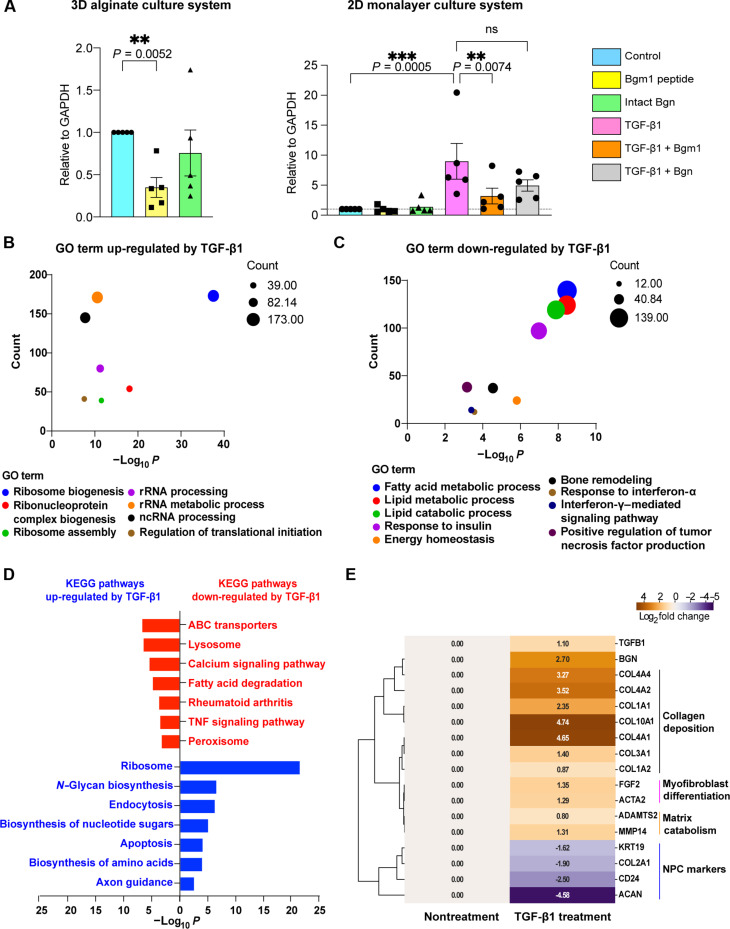
TGF-β1 treatment induces a degenerative change in human NPCs. (**A**) *COL1A1* expression changes after 14 days of Bgm1 peptide treatment in 2D monolayer and 3D alginate culture systems in hND-NPCs from adolescent patients undergoing scoliosis correction surgery. Both culture systems consisted of five patients each. mRNA expression of all the groups was normalized with nontreated control. Each dot represents an individual patient. (**B** and **C**) GO term analysis of DEGs significantly regulated by TGF-β1. (**D**) KEGG term analysis of DEGs significantly regulated by TGF-β1. Analysis was achieved via the clusterProfiler package and plotted through Prism 9. The significance was adjusted through *P* < 0.05. (**E**) Representative list showing the changes of IDD-related gene expression under TGF-β1 treatment. The intergroup comparison is TGF-β1 treatment versus nontreatment. Significant DEGs were highlighted in green. *P* < 0.05. Data presented as means ± SD. Statistics were performed by two-way ANOVA. ns, *P* > 0.05; **P < 0.01;***P < 0.001.

**Fig. 4.**
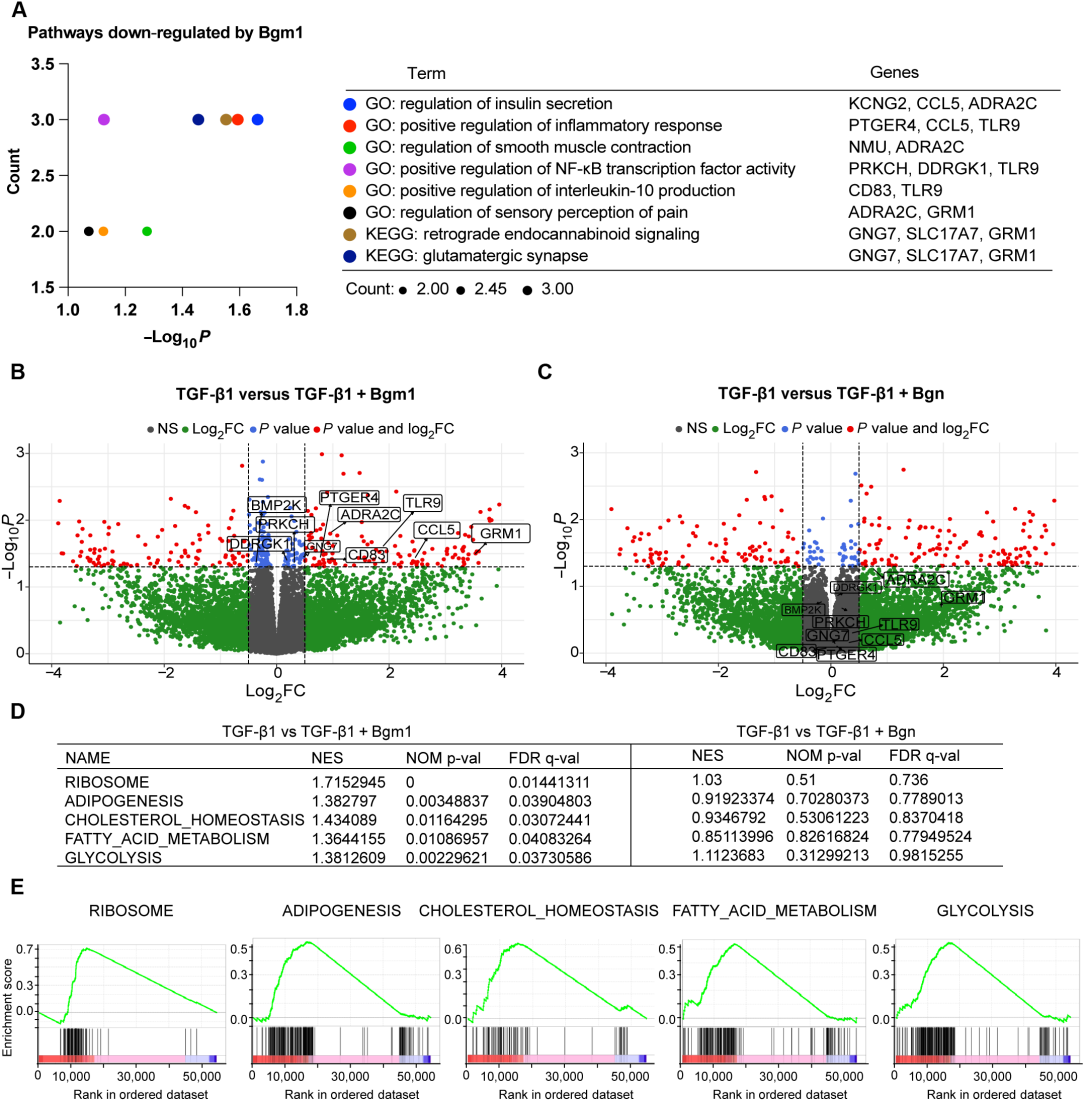
Bgm1 attenuates pathways mediated by TGF-β1 signaling. (**A**) GO term and KEGG analysis of DEGs significantly down-regulated by Bgm1 in the TGF-β1–treated group versus TGF-β1 + Bgm1–treated group. The significantly enriched pathway was adjusted through *P* < 0.05. (**B** and **C**) Volcano plot of DEGs in the TGF-β1 treatment group versus TGF-β1 + Bgm1 treatment group (B) and the TGF-β1 treatment group versus TGF-β1 + Bgn treatment group (C). The *x* axis represented the fold change, and the *y* axis represented significance. Log_2_ fold change (FC) cutoff 0.5 and *P* value cutoff 0.05 were used. TGF-β1 + Bgm1 induced a negative log_2_ FC for up-regulated genes. The red color on the upper right represents genes significantly down-regulated by Bgm1, and the green area represents genes with nonsignificant expression. (**D**) Gene sets significantly enriched among class A (TGF-β1–treated group) and class B (TGF-β1 + Bgm1 treatment group) (left panel); gene sets not significantly enriched among class A (TGF-β1–treated group) and class B (TGF-β1 + Bgn treatment group) (right panel). (**E**) GSEA distribution graph of Bgm1 under TGF-β1 treatment. Class A: TGF-β1–treated group; class B: TGF-β1 + Bgm1 treatment group. The green curve represented the enrichment score of each gene corresponding to the gene set, the barcode-like black lines were the locations of the genes in the gene set, and each vertical line represented a gene in that pathway. The enriched gene sets showed an increasing trend. Significant enrichment criterion: |NES| > 1; p-val < 0.05; FDR q-val < 0.25.

Transforming growth factor–β1 (TGF-β1) signaling is essential to normal development of IVDs. However, overactivation of TGF-β signaling can contribute to the progression of IDD by influencing the matrix content, cell viability, and inflammatory responses (*33*). TGF-β1 dysregulation is also associated with various fibrotic diseases (*34*). Since biglycan can modulate TGF-β activity (*35*), we investigated the impact of exogenous Bgm1 on the expression of fibroblastic (*FAP*, *FSP*, and *COL1A1*) and chondrogenic (*COL2A1*, *SOX9*, and *ACAN*) markers in the hND-NPCs in the presence of TGF-β1 signaling (fig. S4). We compared the effect between monolayer and alginate culture systems, which may differentially influence the NPC phenotype (*36*). Results showed that Bgm1, but not full-length biglycan, significantly down-regulated *COL1A1* expression in committed NPCs and inhibited the TGF-β1–mediated up-regulation of *COL1A1* expression (Fig. 3A). Transcriptome analysis showed that TGF-β1 up-regulated genes related to translational initiation, ribosome biosynthesis, and glycan biosynthesis (Fig. 3, B and D). It down-regulated genes in pathways pertinent to lipid metabolism, particularly fatty acid catabolism, consistent with the reported negative impact of increased lipid level in IDD (Fig. 3, C and D) (*37*). TGF-β1 treatment also up-regulated genes implicated in collagen deposition, myofibroblast differentiation, and matrix catabolism along with concurrent down-regulation of NPC markers (Fig. 3E). Addition of exogenous Bgm1 in hND-NPCs could down-regulate TGF-β1–triggered pathways related to insulin secretion, inflammatory responses, and pain perception (Fig. 4A). This included a reduced expression of inflammation-related genes *PTGER4*, *CCL5*, *TLR9*, *DDRGK1*, and *CD83*, as well as pain-sensation genes *GNG7*, *SLC17A7*, *GRM1*, and *ADRA2C*; in contrast, full-length biglycan exhibited no notable modulatory effects (Fig. 4, B and C). Gene set enrichment analysis (GSEA) indicated that Bgm1, but not full-length biglycan, could down-regulate ribosome biosynthesis in addition to metabolic pathways encompassing adipogenesis, glycolysis, cholesterol homeostasis, and fatty acid metabolism induced by TGF-β1 (Fig. 4, D and E). Comparing both peptide treatments under TGF-β activity revealed that Bgm1 could down-regulate inflammatory genes (*TLR1* and *CD83*) (fig. S5A) and up-regulate transcription- and cell cycle–related pathways (fig. S5B). GSEA also supported Bgm1-mediated suppression of lipid metabolism and inflammation (fig. S5C).

### Bgm1 interacts with eIF6

**Fig. 5.**
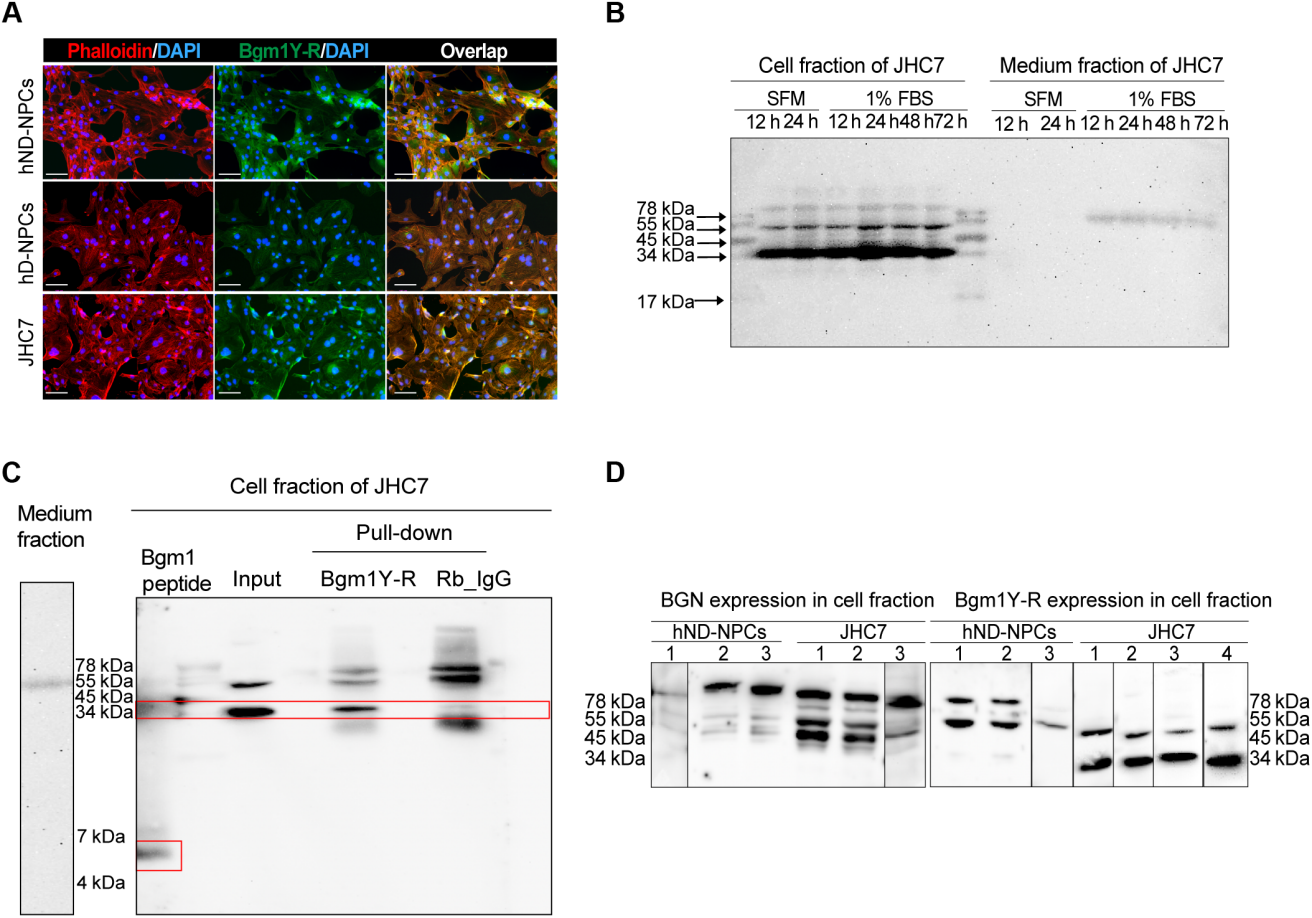
Bgm1 expression in human NPCs and JHC7 cells. (**A**) Representative immunofluorescence images of Bgm1 expression in hND-NPCs, hD-NPCs, and JHC7 cells. (**B**) Representative images of Western blots showing differential intracellular and extracellular expression of Bgm1 under different conditions (SFM: 12 and 24 hours; 1% FBS: 12, 24, 48, and 72 hours) in JHC7 cells. SFM, serum-free medium; 1% FBS, serum reduction medium containing 1% FBS. (**C**) Bgm1 identification after Bgm1Y-R pull-down in JHC7 cells. The synthetic Bgm1 peptide was used to show the Bgm1 molecular weight. The total protein lysate was used as input control, and rabbit IgG (Rb_IgG) was used as a negative control for the Bgm1Y-R antibody. The red boxes highlighted the bands of specific expression of the Bgm1 peptide and Bgm1Y-R pulled down. (**D**) Western blot analysis of BGN and Bgm1 in hND-NPCs and the JHC7 cell line. Each cell type had at least three biological replicates labeled as hND-NPC_1,2,3 and JHC7_1,2,3,4. Scale bars, 100 μm.

The primitive mouse and human NP consists of mainly notochordal cells that can transform into chondrocyte-like cells in IDD (*7*, *38*). Our staining data supported an association of Bgm1 with the primitive NPCs. We therefore hypothesized a preferential expression and function of Bgm1 in the notochordal cells. In JHC7 cells, a widely used notochordal cell model, we detected strong cytosol expression of Bgm1 (Fig. 5A) and identified a primary Bgm1 signal at ~34 kDa in Western blot (Fig. 5B). Immunoprecipitation study confirmed the expression in the cellular fraction (Fig. 5C). Signals were found at lower molecular weights in JHC7 cells (minimum of 34 kDa) compared to those in hND-NPCs (minimum of 55 kDa) (Fig. 5D). Given the predicted size of the Bgm1 peptide (~5 kDa), we hypothesized that the Bgm1Y-R antibody recognized a complex in which Bgm1 coupled to and regulate the activity of other proteins in the notochordal cells.

**Fig. 6.**
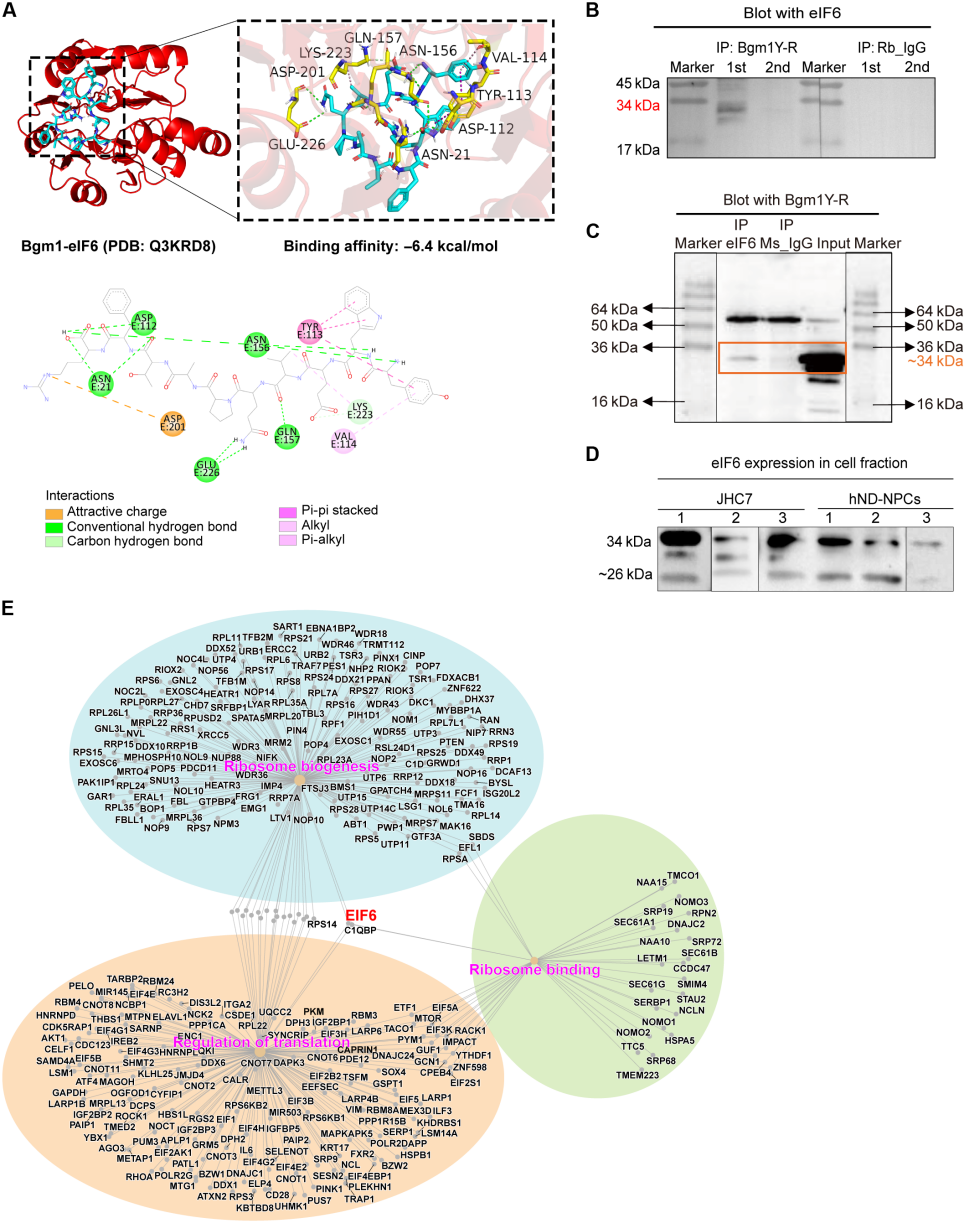
Identification of eIF6 as a binding target of Bgm1. (**A**) Intermolecular interactions between Bgm1 and eIF6. The Bgm1 peptide (in blue) and eIF6 protein (PDB: Q3KRDB, in red) docking result is visualized using PyMOL2.3.0 software and Discovery Studio2019. The different forces and binding modes resulting from docking are indicated by different colored dashed lines and explained by type below the 2D diagram. (**B**) Coimmunoprecipitation of Bgm1-eIF6 interaction in JHC7 cells. The Bgm1Y-R pull-down lysate was detected by the eIF6 antibody at ~34 kDa. 1st and 2nd, two separate elutions of the immunoprecipitated magnetic beads. (**C**) Coimmunoprecipitation of Bgm1-eIF6 interaction in JHC7 cells. The eIF6 pull-down lysate was detected by the Bgm1Y-R antibody at ~34 kDa. (**D**) Representative images of Western blots showing eIF6 production in hND-NPCs and the JHC7 cell line. Each type of cell underwent biological triplicates labeled as hND-NPC_1,2,3 and JHC7_1,2,3. (**E**) Net plot of the RNA sequencing data visualizing a connection of *EIF6* to the ribosome- and translation-related pathways up-regulated by TGF-β1. Each color represents a single GO term, and the dot size signifies the involved gene number. IP, immunoprecipitation.

Analysis of the ~34-kDa band by liquid chromatography–tandem mass spectrometry (LC-MS/MS) identified three candidate proteins, with eIF6 showing the highest score (table S3). Docking analysis substantiated an interaction between Bgm1 and eIF6 with a high binding affinity (−6.4 kcal/mol), in which Bgm1 forms eight conventional hydrogen bonds, a carbon-hydrogen bond, and π-π stacking and π-alky interactions with eIF6 (Fig. 6A). Coimmunoprecipitation study in JHC7 cells using reciprocal antibodies showed that an eIF6-containing complex could be identified in Bgm1 pull-down and vice versa (Fig. 6, B and C), validating the interaction. Upon examining eIF6 production in both JHC7 cells and human NPCs, we detected a putative Bgm1-eIF6 complex at ~34 kDa in addition to its documented molecular weight (26 kDa) (Fig. 6D). Our transcriptome analysis also identified *EIF6* as a central gene shared among the ribosome- and translation-related pathways up-regulated by TGF-β1 (Fig. 6E), supporting a modulatory effect of Bgm1 on TGF-β1 signaling via eIF6.

**Fig. 7.**
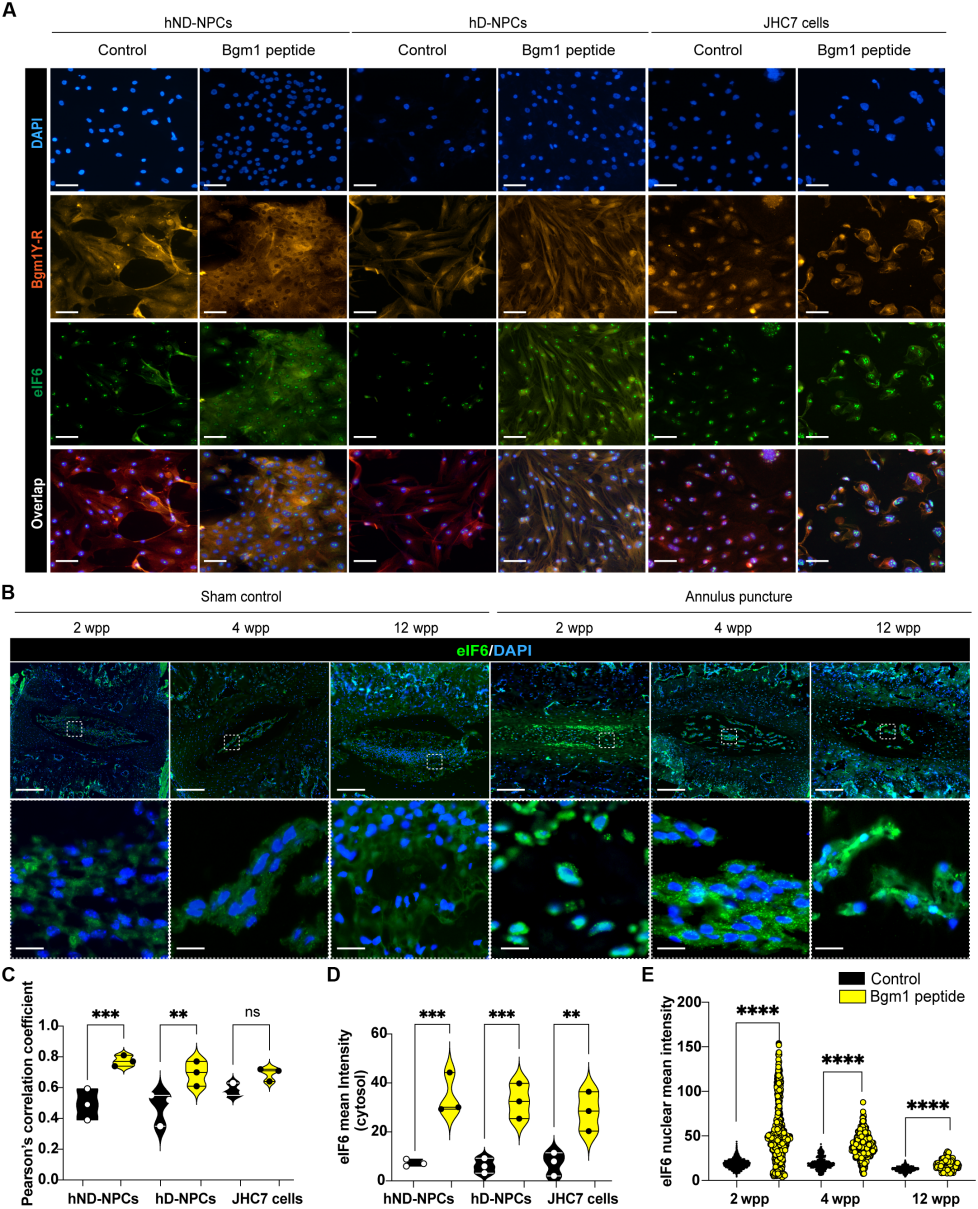
Localization of eIF6 in response to the presence of Bgm1. (**A**) Representative immunofluorescence image of Bgm1 and eIF6 expression with and without 24 hours of Bgm1 peptide treatment in hND-NPCs, hD-NPCs, and JHC7 cells. Scale bars, 100 μm. (**B**) Representative immunofluorescence image of eIF6 with and without annulus puncture–induced mouse IDD models. (**C**) The statistics of colocalization coefficients between Bgm1 and eIF6 are shown in violin plots. Each dot represents a biological replicate for each cell line. (**D**) Quantitative results of eIF6 cytoplasmic expression in response to Bgm1 treatment. Each dot represents a biological replicate for each cell line. (**E**) Nuclear eIF6 expression level in IVD at 2, 4, and 12 wpp. Data were quantified from five different fields of each sample. Each dot represents each cell. Number of quantified cells in each group: 2 wpp sham = 1531; puncture = 658; 4 wpp sham = 509; puncture = 465; 12 wpp sham = 746; puncture = 334. The *P* value was calculated by two-tail unpaired Mann-Whitney test. Scale bars, 10 and 100 μm. Data presented as means ± SD. Unpaired two-tailed Student *t* test was used for a two-group comparison (ns, *P* > 0.05; **P < 0.01; ***P < 0.001; ****P ≤ 0.0001).

eIF6 is a key translational initiation factor that regulates ribosomes synthesis, cell proliferation, and gene expression (*39*). It is translocated from the nucleus to the cytosol during ribosome maturation (*39*). In the nucleus, eIF6 is necessary for ribosomal 60S subunit biogenesis, while in the cytoplasm, eIF6 is required for insulin regulation through its translational activity (*40*). Immunostaining verified a predominant nuclear expression of eIF6 in both JHC7 cells and NPCs (Fig. 7A). We speculated that the eIF6 localization, through interaction with Bgm1, may be dysregulated by supplying exogenous Bgm1. As expected, treating the cells with the Bgm1 peptide induced cytosolic expression of eIF6 (Fig. 7D) in addition to its nuclear expression (fig. S6). Signal correlation analysis validated intensified Bgm1 and eIF6 colocalization (Pearson correlation coefficient exceeding 0.5) (Fig. 7C) (*41*). These findings therefore indicate a function of Bgm1 in inducing the expression of and sequestering eIF6 in the cytosol. Moreover, examination of the expression of eIF6 in the puncture-induced degenerative mouse discs (Fig. 7B) revealed a significant increase in nuclear eIF6 expression in the NP compared to the healthy discs (Fig. 7E). These results therefore indicate that the interaction between Bgm1 and eIF6 facilitates the translocation and sequestration of eIF6 in the cytosol and that the loss of Bgm1 in the course of IDD favors a shuffling of eIF6 to the nucleus.

## DISCUSSION

In the present study, we observed distinctive expression patterns for the biglycan neoepitope Bgm1 compared to that of the full protein form. During mouse IVD development, Bgm1 was first detected in the primitive NPC comprising notochordal cells in fetal spine and continued to be produced in the NP and cartilaginous region in the neonatal stage. In contrast, biglycan initiates its production earlier in the notochord, which is later expressed throughout the developing IVDs. In adult mice, Bgm1 is preferentially produced in the NP and the expression is largely reduced in injury-induced IDD, whereas biglycan shows a broader distribution throughout the NP, AF, cartilage endplate, and growth plate. Studies in human NP specimens corroborate the finding of reduced Bgm1 levels in IDD. These suggest that Bgm1 plays a unique role in the NP function, thereby contributing to and serving as a marker of normal IVD homeostasis.

Biglycan is highly abundant in the pericellular matrix of many tissues including bone, cartilage, tendon, and IVD (*42*). In contrast to other SLRPs like decorin and lumican, biglycan was reported to be a preferentially degraded substrate in the early stages of cartilage degradation (*43*). Recently, novel peptides resulting from biglycan fragmentation have been investigated as biomarkers for ECM remodeling–related diseases. For instance, the biglycan neoepitope BGN^262^ (the cleavage site is ^262^GLGHNQIRM) was shown to have a distinct cytoplasmic and intranuclear staining location in osteoarthritic subchondral bone sclerosis (*44*, *45*) and this intracellular expression was more pronounced in osteoarthritis with increasing severity (*44*, *45*). The authors also observed that the molecular weight of the identified BGN^262^ was greater than its theoretical value, but such a discrepancy was not further examined. We propose that this might be related to the complex formation through the Bgm1 peptide at the C terminus. Our docking analysis suggested the presence of a carbon-hydrogen bond and alkyl interactions between Bgm1 and eIF6. Such covalent interactions, particularly those exhibiting high binding energy (ranging from −5.4 to −14.0 kJ/mol), might have conferred strong binding in the complex, which could not be fractionated in SDS–polyacrylamide gel electrophoresis (*46*, *47*). The Bgm1 peptide (YWEVQPATFR) was not identified in the mass spectrometry analysis. This might be due to the binding not being resolved after the in-gel digestion or the absence of the particular Bgm1 sequence in the tryptic digest databases.

**Fig. 8.**
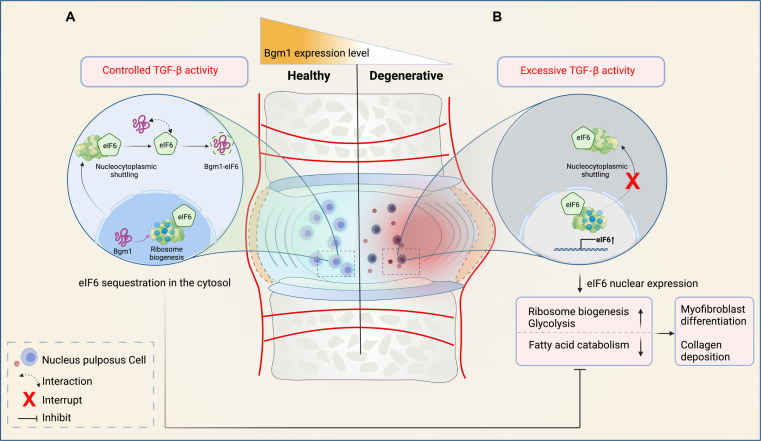
Schematic of IVD homeostasis regulation by Bgm1. Bgm1 is predominantly expressed in healthy NPCs and lost in degenerative NPCs. (**A**) In healthy IVD, Bgm1 facilitates the nucleocytoplasmic shuttling of eIF6 during ribosome biogenesis by interacting with and sequestering eIF6 in the cytoplasm. The interaction between Bgm1 and eIF6 maintains NPC homeostasis by modulating TGF-β signaling at controlled levels. (**B**) In degenerative IVD, excess TGF-β increases eIF6 levels, while the loss of Bgm1 disrupts the nucleocytoplasmic shuttling of eIF6, resulting in a nucleus expression. This leads to the up-regulation of ribosome biogenesis and glycolysis, along with reduced fatty acid catabolism. Consequently, myofibroblast differentiation is promoted, and collagen deposition in NPCs is increased.

eIF6 is essential to ribosome synthesis and regulates the cell translational process (*39*). A small amount of eIF6 can accelerate protein translation, but in abundance, eIF6 can prevent the translation process by restraining ribosome biogenesis (*40*). eIF6 has also been reported to play roles in regulating metabolism and tissue homeostasis. For instance, eIF6 can regulate fatty acid synthesis and glycolysis by modulating *FASN* gene expression in a cell-autonomous manner (*48*). Reprogramming of ribosome biogenesis and metabolism–related pathways has been reported to underlie the EMT process (*49*, *50*). Our data showed that Bgm1 could modify these pathways induced by TGF-β along with a reduction in *COL1A1* expression. In vivo data also indicate a sequestration of eIF6 to cell nuclei in the absence of Bgm1 in the early stage of IDD, which precedes fibrotic remodeling of the NP. Together, these imply that Bgm1 may regulate TGF-β–mediated tissue remodeling by altering the shuttling and, hence, the activity of eIF6 in modifying translation and cell metabolism (Fig. 8). Reports have also suggested that eIF6 can facilitate the inhibition of TGF-β transcription and myofibroblast differentiation and inhibit collagen I protein production induced by physical injury (*51*, *52*). Whether this involves a regulation by Bgm1 awaits further examination. On the other hand, an attenuated eIF6-driven translation can contribute to lipid accumulation in the progression of nonalcoholic fatty liver disease to hepatocellular carcinoma (*53*). Our GSEA analysis also showed that Bgm1 inhibits glycolysis-, adipogenesis-, and fatty acid metabolism–related pathways mediated by TGF-β. This finding further supports a role of Bgm1 in governing cell metabolism and aligns with the reported function of eIF6.

Ribosomes play an important role in proteostasis to maintain cell homeostasis by preventing the production of abnormal or unnecessary proteins. Their synthesis involves coordinated assembly of ribosomal RNA and ribosomal proteins. A role of integrated stress response in musculoskeletal diseases, including IDD, has been reported, which influences the protein folding capacity of the endoplasmic reticulum (*54*, *55*). Our data indicate that TGF-β1 can up-regulate the ribosome biogenesis–related pathway, whereas Bgm1 could inhibit the pathway under TGF-β1 signaling (Fig. 8). This suggests that Bgm1 might also have an impact on proteostasis in IVD.

It is noteworthy that varying sizes of biglycan were observed in the immunoblots. This may reflect the presence of core protein modifications such as glycosylation or cleavage or products that resulted from alternative splicing. A study in skin tissues demonstrated that the fully glycosylated form of biglycan (>140 kDa) is predominantly secreted, while the monoglycosylated (>70 kDa) and nonglycosylated forms (~40 kDa) are found in cell lysates, indicating that various glycosylated forms can coexist (*56*). In contrast, a range of core protein sizes from 36 to 97 kDa could be extracted from articular cartilage after deglycosylation, where fragments below 42 kDa were regarded as degradation products and those above as dimers of biglycan (*57*). In the JHC7 cells, we observed bands ranging from 45 to 105 kDa. Whether they correspond to the monoglycosylated or dimeric forms in addition to the biglycan core protein awaits to be determined. The identification of splice variant 201 of biglycan (ENST00000331595.9), which includes the Bgm1 translation sequence, suggests that these bands might also be related to alternative splicing. On the other hand, we observed localization of Bgm1 staining in the interstitial or pericellular space of the human NP tissues and the postnatal mouse AF. This may reflect an underlying local matrix remodeling event during IVD homeostasis or the maturation process, resulting in biglycan cleavage and accumulation of Bgm1.

A role of nociception has been indicated in IDD-associated back pain (*58*). A previous report suggested that biglycan can reduce the release of substance P from sensory neurons (*59*). We found that Bgm1 treatment in the NPCs could down-regulate pathways related to sensory perception of pain and glutamatergic synapse function under excessive TGF-β activity (Fig. 4A). A comparison to full-length biglycan treatment further showed a substantial inhibitory effect of Bgm1 on nociception-related pathways, including synaptonemal complex assembly and neurotransmitter secretion (fig. S4). This suggests that Bgm1 might also modulate the sensory neuron function and therefore play a regulatory role in disc pain.

A limitation of this study lies in that, while the puncture model can generate degenerative discs that mirror human IDD in terms of morphology and molecular marker expression, it may diverge from the actual degenerative process. For instance, the model involves acute inflammation during the induction stage in contrast to the chronic inflammation observed in IDD (*60*), causing an acute loss of extracellular matrices and structural integrity. This model might therefore favor the discovery of mechanisms related to tissue damage and reparative response. Future study may explore the role of Bgm1 in different IDD models or other biglycan-enriched tissue systems.

In conclusion, our findings indicate that Bgm1 exhibits a distinct expression pattern in the IVD and interacts with eIF6 to engage in the regulation of metabolic and inflammatory pathways. This study implicates a role of the biglycan fragment in spinal health and degeneration. It also implies an alternative intracellular path of SLRP in modulating the TGF-β function.

## MATERIALS AND METHODS

### Human samples

Clinical samples were collected under informed consent and approval by local Institution Review Board. Degenerated and nondegenerative discs were extracted from patients with IDD undergoing spinal fusion surgery and patients with scoliosis undertaking corrective operation, respectively (table S1 for demographics).

### Isolation and culture of human NPCs

Human NPCs were isolated according to our previous protocol (*36*). NPCs were cultured in a complete medium, Dulbecco’s modified Eagle’s medium, high glucose (Gibco, CA), containing 10% fetal bovine serum (FBS), 1% penicillin/streptomycin, 1% l-glutamine, and 0.5% fungizone at 37°C with 5% humidified CO_2_ with the medium refreshed every 2 days. After 1-week culture, cobblestone-like cells were observed. Three-dimensional (3D) alginate culture of human NPCs was described in our previous study (*36*).

### Culture of JHC7 cells

JHC7 cells were under 2D monolayer culture in DMEM/F12 (Gibco, CA) medium containing 10% FBS, 1% penicillin/streptomycin, 1% l-glutamine, and 0.5% fungizone at 37°C with 5% humidified CO_2_ with the medium refreshed every 2 days. The cells exhibit an epithelial-like cell phenotype.

### RNA isolation and real-time quantitative polymerase chain reaction

For alginate culture samples, the cells were first released by dissociation buffer (0.05 M sodium dihydrogen citrate and 0.03 M EDTA, pH 6.8) and collected at 300*g*, 5 min after 20-min incubation on ice. Total RNA was extracted using the RNeasy Mini Kit (Qiagen, Germany). One thousand nanograms of RNA was used for the synthesis of cDNA using the PrimeScript RT Reagent Kit (Takara) with the standard protocol. The synthetic cDNA was mixed with mastermix in the GoTaq qPCR System Kit (Promega) and performed real-time polymerase chain reaction (PCR) by a QuantStudio 1 Real-Time PCR System (Thermo Fisher Scientific). Relative gene expression levels were analyzed by using the 2^−ΔΔ*C*t^ method. The target expression was normalized with the housekeeping gene GAPDH (glyceraldehyde-3-phosphate dehydrogenase). The primer sequences of a specific gene are listed in the table S2.

### Bgm1 antibody generation and specificity test

The YWEVQPATFR (Bgm1) sequence was submitted to Thermo Fisher Scientific for the generation of a custom rabbit polyclonal antibody (Bgm1Y-R). A cysteine residue and lysine residue were added to the N terminus of the provided peptide sequence to enhance the conjugation and solubility of the antibody during production. The specificity of the Bgm1Y-R antibody was validated through immunofluorescence staining performed on 293 cell lines with biglycan knockout. The biglycan knockout cell line was generated using CRISPR-UTM–mediated genome engineering by Ubigene Biosciences. The guide RNA sequence that targets biglycan is CAGAGACACGAGGCGCCACA GGG. The anti-biglycan antibody (Abcam, ab231297, anti-rabbit) was used as staining control, which recognizes an immunogen corresponding to the C-terminal 200 amino acids of human biglycan.

### Protein extraction, immunoprecipitation, gel staining, and Western blotting

Every 1 ml of radioimmunoprecipitation assay buffer with protease inhibitors and phosphate Stop (Roche) was added into 10^7^ cells for cell fraction extraction. The phenol red–free medium containing secreted protein was directly collected after 12, 24, 48, or 72 hours of culture in serum-free medium (SFM) or serum-reduced medium (containing 1% FBS) for medium fraction. The protein concentration was measured by the Bradford Assay Kit (Thermo Fisher Scientific). One milligram of protein lysate per reaction was submitted for the pull-down assay. Immunoprecipitation was conducted using a custom anti-Bgm1 antibody (Thermo Fisher Scientific) and the Protein A/G Mixed Magnetic Bead System (PureProteome, HK), following the manufacturer’s protocol (*61*, *62*). Protein samples (at least 20 μg per well) were fractionated by electrophoresis in 16% tricine SDS gel and transferred to a polyvinylidene difluoride membrane at 180 mA for 1 hour. The membrane was blocked with 5% bovine serum albumin in Tris-buffered saline with 0.1% Tween 20 detergent (TBST) overnight at 4°C; incubated with custom anti-Bgm1 (Bgm1Y-R, anti-rabbit, 1:500), anti-eIF6 (ab124839, anti-rabbit, 1:1000), anti-biglycan (ab231297, anti-rabbit 1:200), and anti-eIF6 (sc390432, anti-mouse, 1:500) at 4°C overnight; and combined with VeriBlot for IP Reagent [horseradish peroxidase (HRP)] (ab131366, 1:1000), anti-mouse HRP (Cell Signaling, catalog no. 91196, 1:1000), and anti-rabbit HRP (Cell Signaling, catalog no. 7074, 1:1000) for 2 hours at room temperature. The result was visualized using Clarity Western ECL Substrate (Bio-Rad) under a Thermo Fisher Scientific chemiluminescence photoimager.

To visualize the band distribution of protein, the additional gel was stained by Coomassie R-250 (Thermo Fisher Scientific). Images were captured on a white stage with light. The interested protein band was excised and processed for tryptic in-gel digestion and LC-MS/MS analysis.

### Liquid chromatography–tandem mass spectrometry analysis

Liquid chromatography–tandem mass spectrometry (LC-MS/MS) was performed and analyzed by the Centre for PanorOmic Sciences, the University of Hong Kong. Protein identification was conducted using an online reverse-phase nanoLC system coupled to an Orbitrap Fusion Lumos mass spectrometer. The SEQUEST search engine was used to analyze the acquired MS/MS spectra, and subsequent protein identifications were subjected to further validation using Proteome Discoverer software. Statistical analyses were used to enhance the confidence of peptide identifications. Confidence criteria (unique peptide ≥1; score, >6.1; MS/MS count ≥2) were used to screen out the protein detected.

### Peptide-protein molecular docking

The docking analysis of eIF6 was conducted by referencing the human amino acid sequence and the corresponding 3D structure [Protein Data Bank (PDB) ID: Q3KRD8]. The molecular configuration of Bgm1 was constructed using ChemDraw software, and subsequent optimization through Chem3D ensured the attainment of a conformation with favorable energetic properties. Before the docking simulations performed using PyMOL 2.3.0 software, unnecessary protein chains, water molecules, and protoligands were removed from the protein structure. Small molecules were hydrogenated using AutoDock Tools 1.5.6 to identify distortable bonds. A grid board was used to define the docking range, using a semiflexible docking approach in conjunction with the Lamarckian genetic algorithm. The final docking outcomes, including binding affinities and related results, were systematically extracted from AutoDock Vina 1.2.0 and Discovery Studio software platforms.

### Immunocytochemistry

JHC7 cells and human NPCs were seeded at a density of 3 × 10^4^ per well on an eight-well chamber slide. The cells were fixed with 4% paraformaldehyde for 15 min. Permeabilization was performed using 0.5% Triton X-100 for 5 min. The cells were blocked with 5% bovine serum albumin for 30 min and incubated with primary antibody anti-eIF6 (Santa Cruz Biotechnology, 1:100) and custom Bgm1Y-R antibody (Thermo Fisher Scientific, 1:100) at 4°C overnight. The cells were then incubated with secondary antibody goat anti-rabbit immunoglobulin G (IgG; Alexa Fluor 555, Thermo Fisher Scientific, 1:500), Donkey anti-Mouse IgG (Alexa Fluor 488, Thermo Fisher Scientific, 1:500), and Alexa Fluor 555 Phalloidin (Thermo Fisher Scientific, 1:100) for 60 min in the dark. The samples were mounted with Hardset Antifade Mounting Medium with 4′,6-diamidino-2-phenylindole (DAPI) and visualized under a Nikon Eclipse 80i Compound Fluorescent Microscope.

### Bgm1 uptake study

The JHC7 cells were seeded into an eight-well chamber slide at a density of 3 × 10^4^ per well. FITC-Bgm1 (Royo, China) was fully dissolved in UltraPure DNase/RNase-Free Distilled Water (Thermo Fisher Scientific) before use. Then, the JHC7 cells were treated with FITC-Bgm1 in phenol red–free medium (1 μg/ml) and FITC-Bgm1 was added every 2 days during medium refresh. The cells were incubated at 37°C with 5% humidified CO_2_, avoiding light. The cellular nucleus was detected using Hoechst 33342 according to the ER Staining Kit (Abcam). The image was captured after 1, 3, and 5 days.

### Mouse models and histological analysis

The animal experiment protocols were approved by local government agency (Department of Health, Hong Kong SAR) and ethics committee (Committee on the Use of Live Animals in Teaching and Research). Developing C57/6J mice at various stages, including E12.5, E14.5, E15.5, and P15, representing the developmental progression from the notochord to the NP (*7*), were harvested for histological analysis. An annulus puncture–induced IDD model was established in 8-week-old male C57/6J mice, as described in our previous study (*7*). The disc tissue sections were obtained at 2, 4, 8, and 12 wpp. The fresh tissues were fixed in 4% paraformaldehyde at 4°C overnight, decalcified in Morse’s solution at 4°C overnight, paraffin embedded, and sectioned at a thickness of 5 μm. Hematoxylin and eosin (H&E) staining was performed according to routine protocols (*63*, *64*). FAST staining was performed as described in our previous study (*28*). Picrosirius red (PSR) staining was performed on the basis of the protocol from J. A. Kiernan (London, Canada) (*65*).

For immunostaining, the sections were incubated with 0.8% hyaluronidase (Sigma-Aldrich) at 37°C for 1 hour before being incubated with anti-biglycan primary antibodies. For eIF6 staining, the unmasking method is 10-min incubation with proteinase K solution (20 μg/ml) in tris-EDTA buffer. After antigen retrieval, the sections were washed with phosphate-buffered saline and blocked with protein block solution (DAKO), followed by primary antibody incubation. On the second day, appropriate secondary antibodies were added and incubated at room temperature for 1.5 hours. After being washed with phosphate-buffered saline for three times, the sections were mounted with a drop of mounting DAPI medium and visualized under a Nikon Eclipse 80i Fluorescent Microscope. For immunohistochemistry staining, the human NP sections were processed with the ImmPRESS Duet Double Staining Polymer Kit (VECTOR). The counterstaining procedure was performed according to the Methyl Green Counterstain kit (VECTOR). Bgm1Y-R–positive staining was quantified using a conventional semiquantitative analysis based on Fiji (ImageJ) (*66*). Expression levels were determined by calculating the percentage of Bgm1Y-R–positive areas relative to the total sample area. Human NP sections were derived from three patients for each of the degenerative and nondegenerative group.

### Colocalization analysis and eIF6 subcellular expression analysis

Colocalization is determined by an algorithm that estimates corresponding coefficients using Pearson’s correlation coefficient. Software packages like Coloc 2 and Colocalization Finder are used for quantitative analysis and estimation of these coefficients. The in vivo eIF6 nuclear expression levels were analyzed using ImageJ. The image was first split into two channels: DAPI channel and eIF6 channel. In the DAPI channel, the threshold in the nuclear channel (DAPI) was adjusted to select nuclear regions of interest (ROIs) with a threshold range of 35 to 255. The watershed function was then applied to separate individual nuclei from their neighboring nuclei. Particle analysis was then performed to detect nucleus boundaries with a minimum size of 15, and the results were added to the ROI manager. In the eIF6 channel, the individual nuclear eIF6 mean intensity was measured on the basis of the ROI list obtained from the DAPI channel analysis. In vitro expression of eIF6 in the nucleus and cytoplasm was obtained by conventional methods using Imaris software version 10.0 (Oxford Instruments, Belfast, UK) (*63*).

### RNA sequencing and analysis

RNA sequencing was performed using hND-NPCs derived from adolescent patients undergoing scoliosis correction surgery and characterized by a lack of Bgm1 expression. RNA sequencing was performed by the Centre for PanorOmic Sciences, the University of Hong Kong. Raw data were routinely processed (*64*). All analyses were carried out in RStudio 1.4.1717. The significance was screened out by *P* < 0.05. The Deseq2 R program was used to normalize the RNA sequencing data and evaluate the differentially expressed genes (DEGs) (*67*). The GO and Kyoto Encyclopedia of Genes and Genomes (KEGG) pathway enrichment analyses were performed by approaching the DEGs into the Database for Annotation, Visualization, and Integrated Discovery version 6.8 web tool and R Studio 4.4.1.

### Statistical analysis

Unpaired two-tailed Student *t* test was conducted to compare data between two groups. Data from more than two groups were analyzed using one-way analysis of variance (ANOVA) (GraphPad Prism 9.0). *P* < 0.05 indicates a statistically significant difference (*P < 0.05, **P < 0.01, ***P < 0.001, and ****P < 0.0001).
