# Supplementary material for: Biglycan fragment modulates TGF-β activity in intervertebral disc via an eIF6-coupled intracellular path
Source: Sci Adv. 2025 Feb 14;11(7):eadq8545. doi: 10.1126/sciadv.adq8545 (PMC11827866; doi:10.1126/sciadv.adq8545)
Supplement: Supplementary file 1 — Figs. S1 to S6 Tables S1 to S4 [file sciadv.adq8545_sm.pdf]

Science Advances
AAAS

Supplementary Materials for

Biglycan fragment modulates TGF-β activity in intervertebral disc via an eIF6-coupled intracellular path

Manyu Zhu *et al.*

Corresponding author: Victor Y. Leung, vicleung@hku.hk

Sci. Adv. **11**, eadq8545 (2025)
DOI: 10.1126/sciadv.adq8545

This PDF file includes:

Figs. S1 to S6
Tables S1 to S4

Supplementary Materials

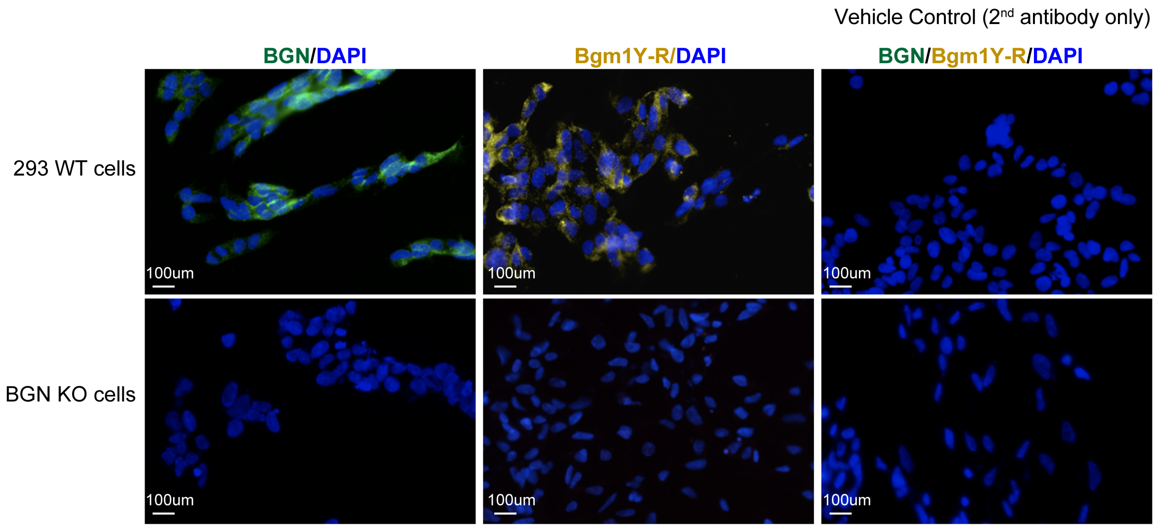

Fig. S1. Bgm1 expression lost in BGN knock out cells

Representative immunofluorescence images of Bgm1 expression in 293 WT and BGN KO cells. BGN KO cells were generated by CRISPR-U^{TM} -mediated genome engineering from Ubigene Biosciences Co.,Ltd. Scale bar:100μm.

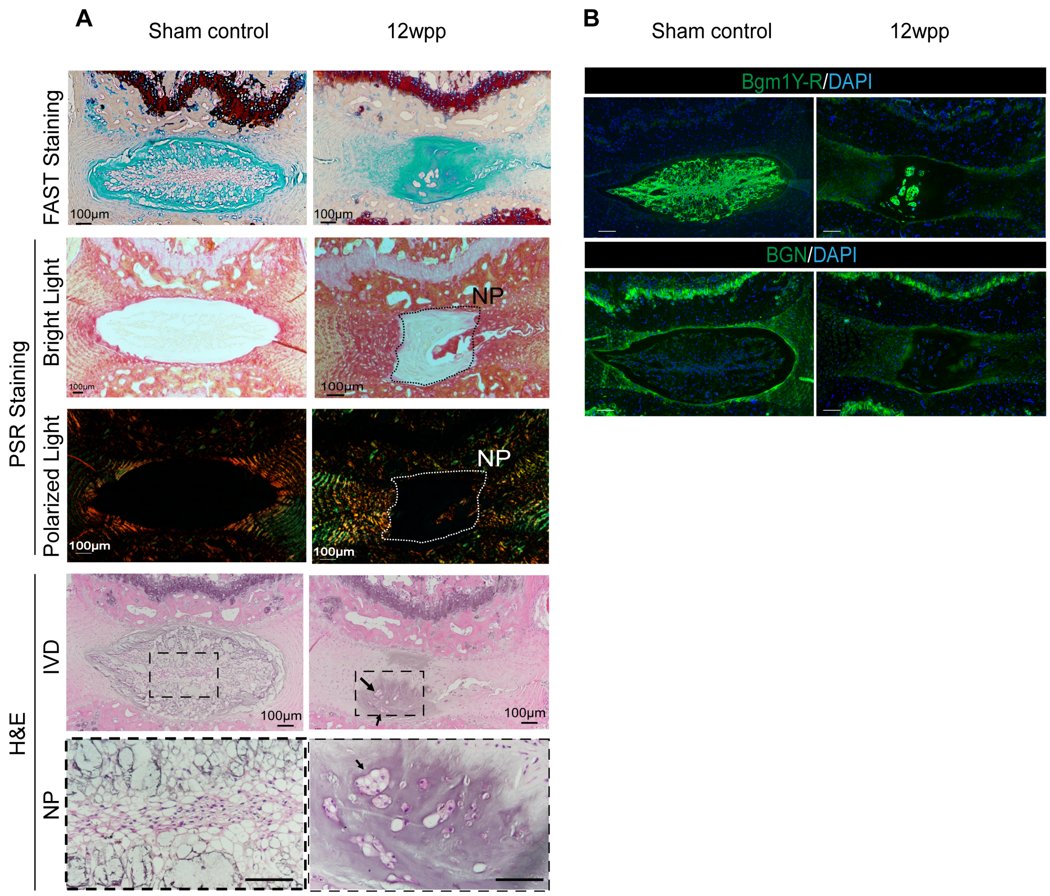

Fig.S2. Bgm1 expression reappeared in NP cell clusters at 12wpp

(A) Representative image of FAST staining, HE staining and Picrosirius Red staining of annulus puncture induced degenerative mouse discs at 12wpp. **(B)** BGN and Bgm1Y-R immunofluorescent staining of annulus puncture induced degenerative mouse discs at 12wpp. Scale bar: 100μm

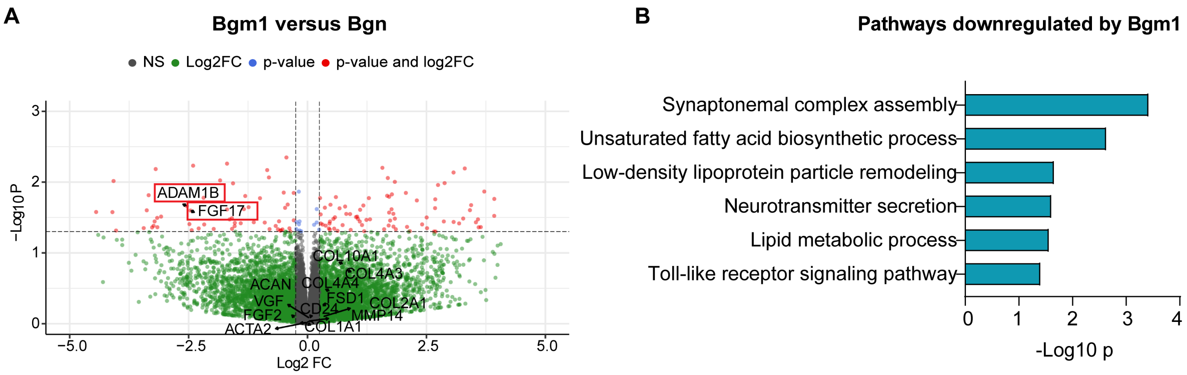

Fig.S3. Functional analysis of Bgm1 compared to full-length Biglycan under controlled TGF-β activity

(A) Volcano plot of DEGs in Bgm1 treatment group versus full-length Biglycan treatment group. Log2FoldChange cut of 0.25, and p-value cut of 0.05. Bgm1 induced a positive Log2FoldChange for upregulated genes. Red box indicated the significant and high fold changed genes under full-length Biglycan treatment. **(B)** GO term analysis of Bgm1 significant downregulated DEGs in the Bgm1 treated group versus full-length Biglycan treated group.

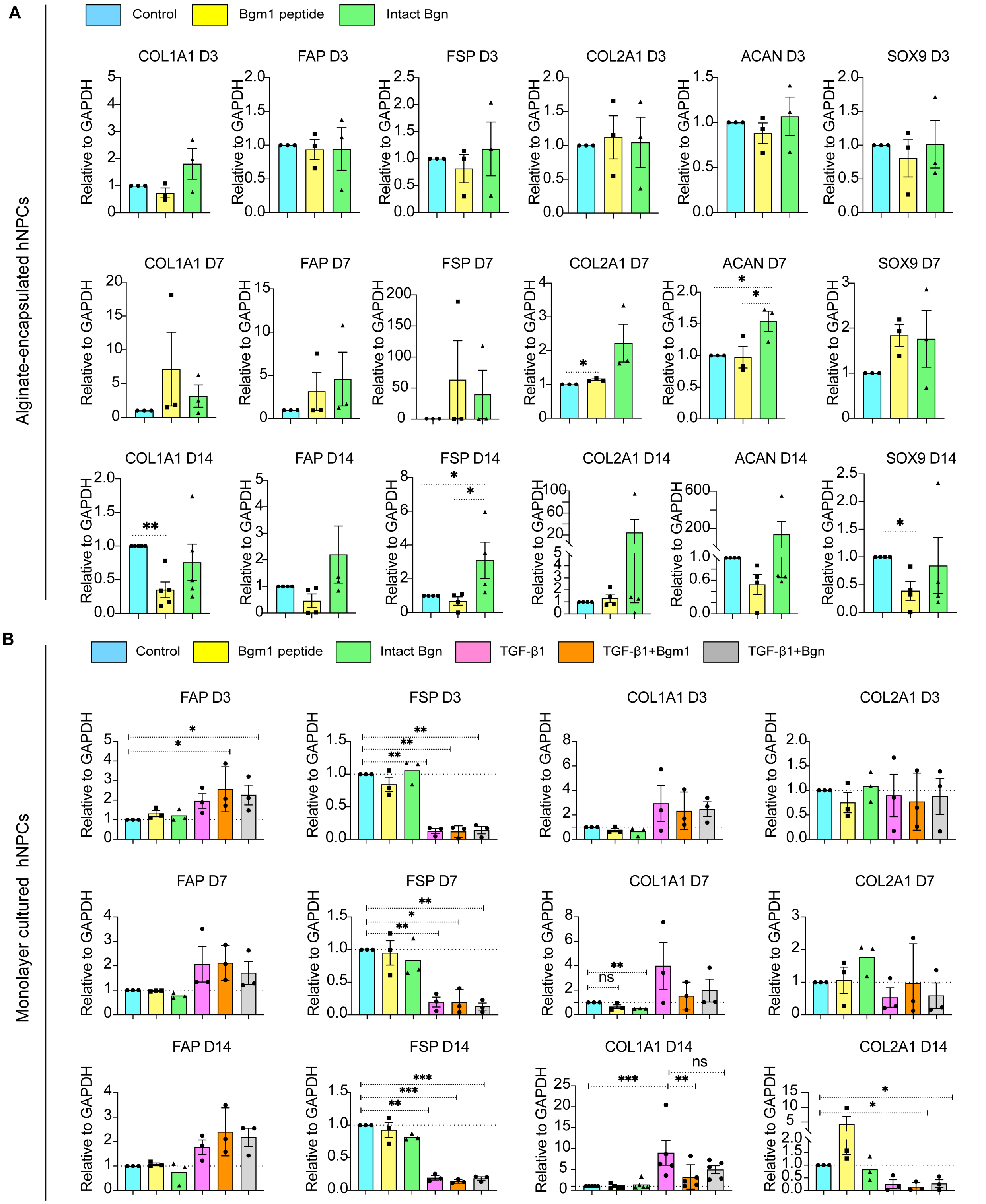

Fig.S4. Changes in fibroblast and chondrocyte markers after Bgm1 treatment *in vitro*

RT-qPCR results of Bgm1 regulated genes in alginate-encapsulated human NP cells (**A**) and monolayer cultured NP cells (**B**). mRNA expression of all the groups were normalized with non-treated control. Data presented as mean ± SD. Statistics were performed by Two-Way ANOVA. (ns, $P > 0.05$; * $P < 0.05$; ** $P < 0.01$; *** $P < 0.001$).

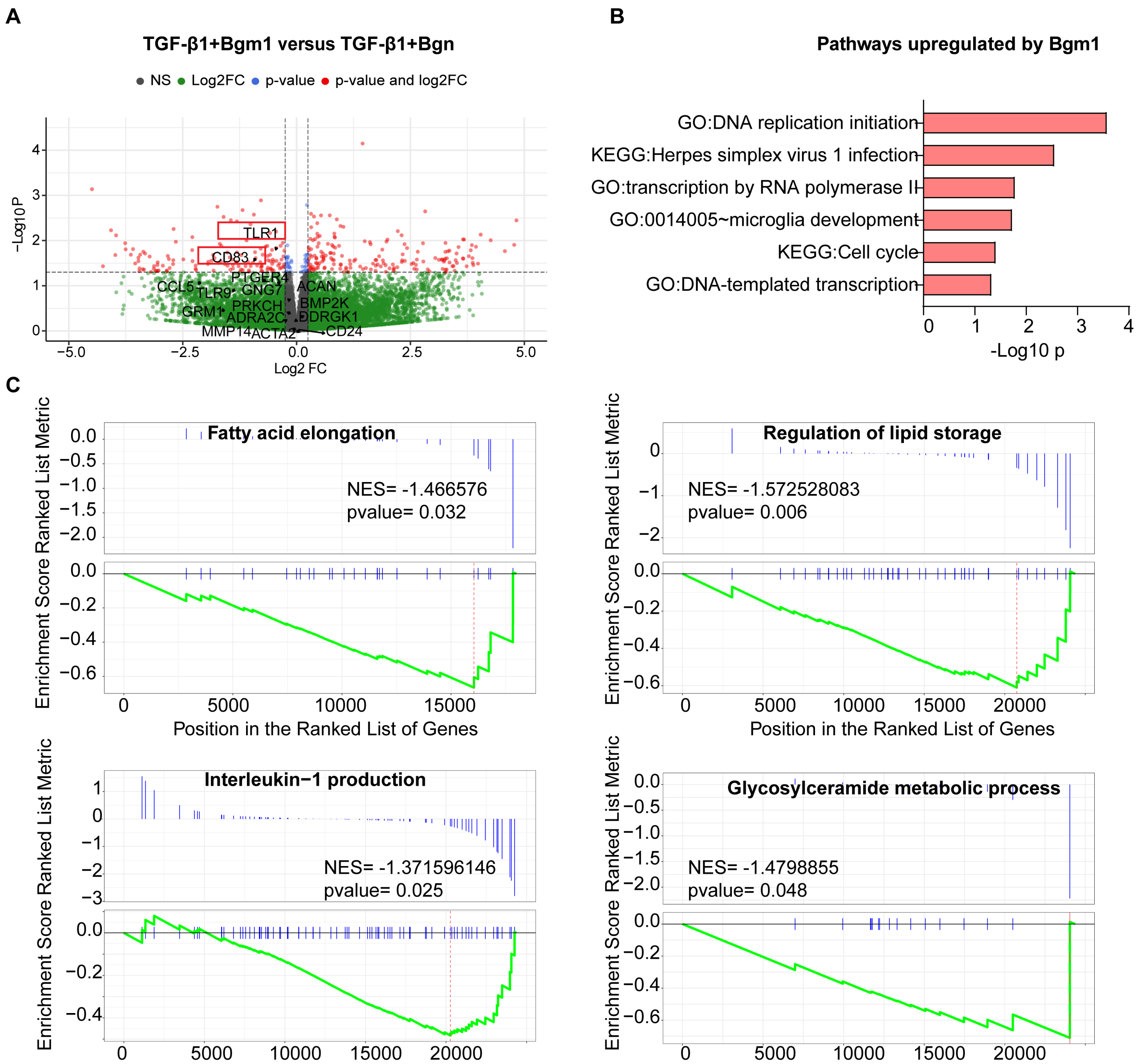

Fig.S5. Functional analysis of Bgm1 compared to full-length Biglycan in TGF-β signaling
(A) Volcano plot of DEGs in TGF-β1+Bgm1treatment group versus TGF-β1+Bgn treatment group. Log2FoldChange cut of 0.25, and p-value cut of 0.05. TGF-β1+Bgm1 induced a positive Log2FoldChange for upregulated genes. Red box indicated the significant and high fold changed genes under TGF-β1+Bgn treatment. **(B)** GO term and KEGG analysis of TGF-β1+Bgm1 significant upregulated DEGs in the TGF-β1+Bgm1 treated group versus TGF-β1+Bgn-treated group. **(C)** GSEA barcode graph of Bgm1 under TGF-β1 treatment. The green curve represented the enrichment score of each gene corresponding to the gene set, the barcode-like black lines showed the locations of the genes in the gene set, and vertical line represented genes in the pathway.

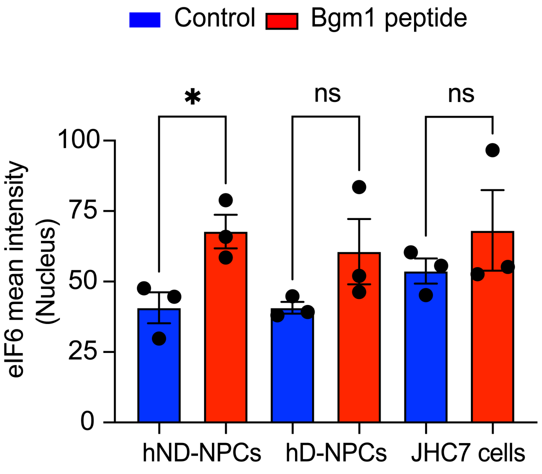

Fig.S6. Quantitative results of eIF6 expression in the nucleus in response to Bgm1 treatment. Each dot represents a biological replicate for each cell line. Data presented as mean ± SD. Unpaired two-tailed Student t-test was used for a two-group comparison. (ns, P > 0.05; * P < 0.05)

	Sample name	Gender	Age	Level	Sore	Application
hND-NP	CDS126	Female	18	L1/2	N/A	RT-qPCR, WB, ICC, RNA-seq
	CDS127	Female	16	L1/2	N/A	RT-qPCR, WB, ICC, RNA-seq
	CDS129	Male	17	T12/L1	N/A	RT-qPCR, WB, ICC, RNA-seq
	CDS124	Female	14	__	N/A	RT-qPCR
	CDS153	Female	47	L2/L3	N/A	RT-qPCR
	CDS102np	__	__	__	N/A	IHC
	CDS103np	__	__	__	N/A	IHC
	CDS96	__	__	__	N/A	IHC
hD-NP	dNP	Male	41	L5-S1	≥IV	ICC
	DS110	Male	50	L4/L5	≥IV	ICC
	dNP112	Female	63	L5/S1	≥IV	ICC
	DS50	Male	58	__	≥IV	IHC
	DS45	Male	48	__	≥IV	IHC
	DS48	Female	45	__	≥IV	IHC

hND-NP: human non-degenerative nucleus pulposus; hD-NP: human degenerative nucleus pulposus
WB: Western Bolt ; ICC: Immunocytochemistry; IHC: immunohistochemistry.

Table.S1. Patient information

**Gene name**		**Sequence**
GAPDH	Forward primer	TCACCACCATGGAGAAGGC
	Reverse primer	GCTAAGCAGTTGGTGGTGCA
COL1A1	Forward primer	GTCACCCACCGACCAAGAAACC
	Reverse primer	AAGTCCAGGCTGTCCAGGGATG
COL2A1	Forward primer	AGGATGGCTGCACGAAACAT
	Reverse primer	GCCATTCAGTGCAGAGTCCT
ACAN	Forward primer	GTGCCTATCAGGACAAGGTCT
	Reverse primer	GATGCCTTTCACCACGACTTC
FSP1	Forward primer	TTCTTGGGGAAAAGGACAGA
	Reverse primer	CTTCCTGGGCTGCTTATCTG
FAPa	Forward primer	TGAACGAGTATGTTTGCAGTGG
	Reverse primer	GGTCTTTGGACAATCCCATGT
SOX9	Forward primer	GCTCTGGAGACTTCTGAACGAGAG
	Reverse primer	CGTTCTTCACCGACTTCCTCC

Table.S2. The primer sequences list

Majority protein IDs	Protein names	Gene names	iBAQ 34kda Bgm1 pull down	iBAQ 34kda IgG	Unique peptides 34kda Bgm1 pull down	Unique peptides 34kda IgG	MS/MS count 34kda Bgm1 pull down	MS/MS count 34kda IgG	Mol. weight [kDa]	Q-value	Score
P56537;B7ZBH1;A0A0U1RQV5	Eukaryotic translation initiation factor 6	EIF6	13.09856	NaN	2	0	2	0	26.599	0	12.387
O00264	Membrane-associated progesterone receptor component 1	PGRMC1	11.26632	NaN	2	0	2	0	21.671	0	11.327
Q9HAV7	GrpE protein homolog 1, mitochondrial	GRPEL1	11.09757	NaN	1	0	2	0	24.279	0	6.3256

- Confidence criteria (Unique peptide ≥ 1, Sore > 6.1; MS/MS count ≥ 2) was used to screen out the protein detected.
- eIF6 showed the highest machine reading scores.

Table.S3 List of proteins identified by LC-MS/MS analysis of ~34kDa specific band pulled down by Bgm1Y-R.

Antibody	Company	Catalog #	Use
Rabbit anti-Bgm1Y-R	Thermo Scientific	custom	IF, IHC, ICC, IP, WB
Rabbit anti-biglycan	Abcam	ab231297	IF, ICC, WB
Mouse anti-eIF6	Santa Cruz	sc390432	IF, ICC, IP, WB
Rabbit anti-eIF6	Abcam	ab124839	IF, ICC, IP, WB
Goat anti-Rabbit AF555	Thermo Scientific	A-21428	IF
Donkey anti-Mouse AF488	Thermo Scientific	A-21202	IF
Alexa Fluor™ 555 Phalloidin	Thermo Scientific	A-31572	IF
VeriBlot for IP Reagent (HRP)	Abcam	ab131366	WB
Goat anti- mouse (HRP)	Cell signaling	91196	WB
Goat anti- rabbit (HRP)	Cell signaling	7074	WB

IF: Immunofluorescent staining; IHC: Immunohistochemistry; ICC: Immunocytochemistry; IP: Immunoprecipitation; WB: Western Bolt

Table.S4. Antibodies used.
